# Higher baseline serum bilirubin levels are associated with increased risk of early neurological deterioration in women with acute ischemic stroke

**DOI:** 10.3389/fneur.2024.1381055

**Published:** 2024-04-08

**Authors:** Meng Sun, Yingfeng Weng, Jiwei Cheng, Guoyi Li, Qian Xiao

**Affiliations:** Department of Neurology, Putuo Hospital, Shanghai University of Traditional Chinese Medicine, Shanghai, China

**Keywords:** bilirubin, ischemic stroke, prognosis, cerebral infarction, clinical deterioration

## Abstract

**Background and objectives:**

Early neurological deterioration (END) occurs in up to one-third of patients with acute ischemic stroke (AIS) and associated with poor outcome. The role of serum bilirubin in END remains controversial. This study aims to investigate the association of total bilirubin (TBIL), direct bilirubin (DBIL) and indirect bilirubin (IBIL) with END.

**Methods:**

This study was a cross-sectional retrospective study with 344 AIS patients enrolled. We retrospectively reviewed consecutive AIS patients with END through a medical record retrieval system and enrolled patients as control randomly from the AIS patients without END at the same period. The bilirubin levels were compared between the END group and No END group. The correlations of bilirubin with END were assessed according to the bilirubin tertiles on the cohort of different genders.

**Results:**

In women, as the bilirubin level increased, the occurrence of END showed an increasing trend. The linear association was significant based on the tertiles of all bilirubin types (TBIL *p* = 0.003; DBIL *p* = 0.025; IBIL *p* = 0.025), while in men no similar trend was observed. After adjustment for confounders, higher TBIL (*p* for trend 0.009) and DBIL (*p* for trend 0.033) levels were associated with increased risk of END in women. The adjusted OR for T3 relative to T1 was 5.240 (95% CI 1.496–18.347) in TBIL and 3.549 (95% CI 1.089–11.566) in DBIL. Multivariate logistic regression showed that DBIL was independently associated with END in women (OR 1.717, 95% CI 1.106–2.666). The study also found that DBIL was superior to TBIL and IBIL in prediction of END occurrence in women, with greater predictive value.

**Discussion:**

There were gender differences in the relationship between bilirubin and END, and DBIL level was positively associated with END occurrence in women, not in men. DBIL had greater incremental predictive value for END than TBIL and IBIL.

## 1 Introduction

Ischemic stroke is a severe cerebrovascular disease with high morbidity, disability and mortality, which places a great burden on the patients and the society. Early neurological deterioration (END) occurs in up to one-third of patients with acute ischemic stroke (AIS) and associated with poor stroke outcome (1). Compared with patients without END, patients with END suffered from higher National Institute of Health Stroke Scale (NIHSS) score at discharge, prolonged hospitalization, and poorer functional outcome. Even a 2-point increase in the NIHSS score was associated with a 3-fold risk of death and was an indicator of poor outcome and in-hospital mortality (2). The treatment of END is still not very satisfactory. Therefore, identifying risk factors associated with END is important for clinically predicting the occurrence of END.

Excessive oxidative stress plays a major role in the pathophysiology of ischemic brain damage in the acute phase of stroke. Human brain is more susceptible to oxidative stress than other organs because of its high consumption of oxygen, abundant unsaturated lipids and relative weak endogenous antioxidant capacity (catalase or glutathione peroxidase) (3). Reactive oxygen (ROS) could damage deoxyribonucleic acid (DNA), cause peroxidation of unsaturated fatty acids in cell membranes, which not only alters cellular integrity, but also leads to reaction with other lipids, proteins and nucleic acids, augmenting damage to the brain (3). Therefore the antioxidant defense system is very important to prevent the brain tissue from ischemia-triggered oxidative stress and cell damage.

Serum bilirubin, the end product of heme metabolism, has been known as the endogenous antioxidant. However, when accumulated highly in tissues, it could also be toxic and cause brain damage especially in newborns. It includes two forms: direct bilirubin (DBIL) and indirect bilirubin (IBIL). IBIL is converted to DBIL by the hepatic enzyme uridine diphosphate glucuronosyltransferase 1A1 (UGT1A1). A number of epidemiological studies showed that moderately high bilirubin was associated with lower risk of cardiovascular disease and mortality (4–6). However, the studies of serum bilirubin in stroke prognosis still remain controversial, without reaching a consensus. Some supported a positive or null relationship between bilirubin and the prognosis of stroke, while others argued that high levels of bilirubin were associated with poor stroke outcomes and mortality. Soleimanpour et al. proposed that bilirubin could be used as a disease predictor and a potential treatment target in stroke, but further researches are required to provide more evidence (7, 8). In addition, there have been a lot of controversies over the gender differences in the relationship of bilirubin with diseases. As far as we know, few studies focused on the relationship of different bilirubin subtypes and END occurrence in different genders.

The objective of our study is to investigate the risk factors of END in AIS, and the association of serum bilirubin subtypes with END occurrence in different genders through a retrospective study.

## 2 Methods

### 2.1 Study population

We retrospectively reviewed consecutive AIS patients with END from January 2019 to June 2021 through a medical record retrieval system of our hospital and enrolled patients as control randomly from the AIS patients without END at the same period. The inclusion criteria were as follows: (1) ≥18 years of age; (2) in compliance with the criteria of Chinese guidelines for the diagnosis and treatment of acute ischemic stroke (9); (3) END was defined as an increase of NIHSS score ≥ 2 points, or an increase of motor subscore ≥ 1 point within 7 days of stroke onset; (4) head CT excluded cerebral hemorrhage. Patients were excluded if they met one of the following criteria: (1) END caused by infection or electrolyte imbalance; (2) diagnosed with all kinds of hepatic diseases; (3) missing data of bilirubin.

### 2.2 Data acquisition

Information on demographic characteristics, medical history and laboratory tests were obtained from the medical record retrieval system by one observer (MS) who was blind to the group assignment. Blood specimens were collected from all subjects within 24 h of hospital admission. Laboratory tests, including total bilirubin (TBIL), direct bilirubin (DBIL), indirect bilirubin (IBIL), total cholesterol (TC), total triglycerides (TG), high-density lipoprotein (HDL), low-density lipoprotein (LDL), lipoprotein a [Lp (a)], glycosylated hemoglobin (HbA1c), high sensitivity C-active protein (hs-CRP), D-dimer, and uric acid (UA), were assessed at the central laboratory of Putuo Hospital affiliated to Shanghai University of Traditional Chinese Medicine. The etiology classification was assessed according to the Trial of Org10172 in Acute Stroke Treatment (TOAST) (10) by two independent observers (YW and JC) and any disagreement was resolved by discussion or consulting other authors (QX and GL) until consensus was reached.

### 2.3 Statistical analysis

Statistical analysis was performed using IBM SPSS Statistics 25.0. Quantitative data in line with normal distribution were expressed as mean ± standard deviation (M ± SD) and compared by the Student’s t test. Quantitative data inconsistent with normal distribution were expressed as the median and interquartile range M (IQR), and compared by non-parametric tests (Mann–Whitney U test). Count data were compared by Pearson Chi-square test. Multivariate stepwise logistic regression was conducted for variables with a *p* < 0.1 on univariate analysis. The predictive power of different bilirubin subtypes adding to model 1 and model 2 was evaluated by Stata 15.0, based on Akaike’s information criterion (AIC), C-statistic, integrative discrimination improvement (IDI) and net reclassification improvement (NRI). Statistical significance was set at *p* < 0.05.

## 3 Results

A total of 344 patients were enrolled in the study: 193 patients in END group and 151 patients in No END group as control. Statistical analysis was conducted on the baseline data of the two groups, and there were significant differences in the proportion of male gender (*p* = 0.046), atrial fibrillation (AF) history (*p* = 0.001), NIHSS score on admission (*p* = 0.048), the levels of UA (*p* = 0.032) and DBIL (*p* = 0.029). No significant differences were found in other factors (shown in Table 1).

**Table 1:** Demographic and baseline characteristics of the No END group and END group.

	No END group	END group	Statistics	*p-*value
*n*	151	193		
Age, year M(IQR)	70 (63–77)	70 (63–78)	Z = −0.016	0.987
Male *n* (%)	96 (63.6)	142 (73.6)	χ^2^ = 3.973	0.046^*^
**Risk factors *n* (%)**				
Hypertension	122 (80.8)	148 (76.7)	χ^2^ = 0.848	0.357
Diabetes	64 (42.4)	91 (47.2)	χ^2^ = 0.777	0.378
Hyperlipidemia	63 (41.7)	94 (48.7)	χ^2^ = 1.665	0.197
Coronary heart disease	17 (11.3)	24 (12.4)	χ^2^ = 0.112	0.738
Smoking history	64 (42.4)	91 (47.2)	χ^2^ = 0.777	0.378
Drinking history	36 (23.8)	50 (25.9)	χ^2^ = 0.193	0.661
Stroke or TIA	38 (25.3)	55 (28.5)	χ^2^ = 0.428	0.513
Atrial fibrillation	17 (11.3)	5 (2.6)	χ^2^ = 10.632	0.001
**TOAST type *n* (%)**			χ^2^ = 6.415	0.093
Large-artery atherosclerosis	43 (28.5)	65 (33.7)		
Cardioembolism	9 (6.0)	3 (1.6)		
Small-artery occlusion	79 (52.3)	106 (54.9)		
Other determined cause	0 (0)	0 (0)		
Undetermined cause	20 (13.2)	19 (9.8)		
**NIHSS score M(IQR)**				
NIHSS score on admission	4 (2–6)	4 (3–6)	Z = −1.981	0.048^*^
Motor score on admission	2 (1–3)	2 (2–4)	Z = −1.941	0.052
**Laboratory tests**				
TC, mmol/L M(IQR)	4.70 (3.84–5.46)	4.78 (4.11–5.61)	Z = −0.945	0.345
HDL, mmol/L M(IQR)	1.05 (0.87–1.21)	1.06 (0.90–1.20)	Z = −0.277	0.782
LDL, mmol/L m ± SD	3.15 ± 0.80	3.26 ± 0.90	t = −1.142	0.254
Lp(a), mg/L M(IQR)	107.00 (54.81–228.50)	90.00 (53.75–169.00)	Z = −1.178	0.239
TG, mmol/L M(IQR)	1.40 (1.08–2.09)	1.44 (1.09–2.00)	Z = −0.013	0.989
HbA1c, % M(IQR)	6.20 (5.80–7.80)	6.20 (5.70–8.00)	Z = −0.244	0.808
hs-CRP, mg/L M(IQR)	1.11 (0.10–3.39)	1.01 (0.10–3.92)	Z = −0.033	0.973
D-dimer, mg/L M(IQR)	0.39 (0.23–0.80)	0.36 (0.20–0.70)	Z = −0.807	0.420
UA, μmol/L m ± SD	339.27 ± 91.53	318.22 ± 87.35	*t* = 2.148	0.032^*^
TBIL, μmol/L M(IQR)	14.00 (10.00–18.00)	14.00 (11.50–19.00)	Z = −1.941	0.052
DBIL, μmol/L M(IQR)	2.40 (1.70–3.20)	2.60 (2.00–3.50)	Z = −2.178	0.029^*^
IBIL, μmol/L M(IQR)	11.50 (8.30–15.00)	12.10 (9.20–15.70)	Z = −1.714	0.086

TIA, transient ischemic attack; TC, total cholesterol; TG, total triglycerides; HDL, high-density lipoprotein; LDL, low-density lipoprotein; Lp(a), lipoprotein a; HbA1c, glycosylated hemoglobin; hs-CRP, high sensitivity C-active protein; UA, uric acid; TBIL, total bilirubin; DBIL, direct bilirubin; IBIL, indirect bilirubin. ^*^p < 0.05. m ± SD, mean ± standard deviation; M(IQR), median (interquartile range, IQR).

We compared the levels of serum bilirubin between different genders and found that the levels of TBIL, DBIL and IBIL were all significantly higher in men than in women (TBIL *p* = 0.008; DBIL *p* = 0.001; IBIL *p* = 0.021). In women, higher levels of bilirubin were observed in END group than No END group, which was not seen in men (shown in Table 2).

**Table 2:** Levels of serum bilirubin in END group and no END group of different genders.

	Women	Men	Statistics	*p-*value^a^
TBIL M(IQR)	13.00 (10.00–16.00)	15.00 (11.00–19.00)	Z = -2.645	0.008^*^
No END	12.00 (10.00–15.00)	15.00 (10.25–19.00)		
END	13.00 (12.00–18.00)	15.00 (11.00–19.25)		
*p*-value^b^	0.033^*^	0.592		
DBIL M(IQR)	2.15 (1.60–2.80)	2.70 (1.90–3.63)	Z = -3.326	0.001^*^
No END	2.00 (1.60–2.70)	2.60 (1.73–3.68)		
END	2.40 (1.90–3.15)	2.73 (2.00–3.63)		
*p*-value^b^	0.078	0.368		
IBIL M(IQR)	10.55 (8.68–14.10)	12.20 (8.88–15.86)	Z = −2.312	0.021^*^
No END	9.60 (8.00–12.10)	12.10 (8.43–15.96)		
END	11.40 (9.50–15.30)	12.28 (9.00–15.83)		
*p*-value^b^	0.037^*^	0.717		

^a^Women vs. men; ^b^No END group vs. END group. ^*^p < 0.05. M(IQR), median (interquartile range, IQR).

The clinical outcomes were compared according to the bilirubin tertiles on the cohort of different genders -respectively, -and different trends were found in women and men. In women, as the bilirubin level increased, the occurrence of END showed an increasing trend (TBIL *p* = 0.003; DBIL *p* = 0.025; IBIL *p* = 0.025). The proportion of END was significantly higher in the 3rd tertile (T3) than in the 1st tertile (T1) in women according to the TBIL (*p* = 0.003), DBIL (*p* = 0.024) and IBIL tertiles (*p* = 0.024). In men, there was no such trend (shown in Table 3).

**Table 3:** The clinical outcomes according to bilirubin tertiles.

Tertiles		No END *n* (%)	END *n* (%)	Statistics	*p*-value^a^	Statistics	*p*-value^b^
**Women**							
TBIL	T1	26 (68.4)	12 (31.6)	χ^2^ = 8.856	0.003^*^	χ^2^ = 8.948	0.003^*^
	T2	19 (51.4)	18 (48.6)				
	T3	10 (32.3)	21 (67.7)				
DBIL	T1	25 (69.4)	11 (30.6)	χ^2^ = 5.024	0.025^*^	χ^2^ = 5.100	0.024^*^
	T2	15 (42.9)	20 (57.1)				
	T3	15 (42.9)	20 (57.1)				
IDBL	T1	24 (66.7)	12 (33.3)	χ^2^ = 5.024	0.025^*^	χ^2^ = 5.073	0.024^*^
	T2	17 (48.6)	18 (51.4)				
	T3	14 (40.0)	21 (60.0)				
**Men**							
TBIL	T1	35 (43.8)	45 (56.3)	χ^2^ = 0.107	0.744	χ^2^ = 0.082	0.774
	T2	32 (36.4)	56 (63.6)				
	T3	29 (41.4)	41 (58.6)				
DBIL	T1	33 (40.7)	48 (59.3)	χ^2^ = 0.124	0.724	χ^2^ = 0.128	0.720
	T2	33 (42.3)	45 (57.7)				
	T3	30 (38.0)	49 (62.0)				
IDBL	T1	34 (42.0)	47 (58.0)	χ^2^ = 0.037	0.848	χ^2^ = 0.036	0.850
	T2	30 (38.5)	48 (61.5)				
	T3	32 (40.5)	47 (59.5)				

Women: TBIL: T1: ≤11 μmol/L; T2: 11–15 μmol/L; T3: >15 μmol/L; DBIL: T1: ≤1.8 μmol/L; T2: 1.8–2.5 μmol/L; T3: >2.5 μmol/L; IBIL: T1: ≤9.4 μmol/L; T2: 9.4–12 μmol/L; T3: >12 μmol/L. Men: TBIL: T1: ≤12 μmol/L; T2: 12–18 μmol/L; T3: >18 μmol/L; DBIL: T1: ≤2.1 μmol/L; T2: 2.1–3.2 μmol/L; T3: >3.2 μmol/L; IBIL: T1: ≤10.2 μmol/L; T2: 10.2–14.5 μmol/L; T3: >14.5 μmol/L; ^a^linear-by-linear association; ^b^T3 vs. T1; ^*^p < 0.05.

We further explored the association of bilirubin with the occurrence of END (shown in Table 4). After adjustment for age, NIHSS on admission, hypertension, diabetes, hyperglycemia, AF, smoking, TOAST types, UA, HDL and LDL, higher TBIL (*p* for trend 0.009) and DBIL (*p* for trend 0.033) levels were associated with increased risk of END in women. The adjusted OR for T3 relative to T1 was 5.240 (95% CI 1.496–18.347) in TBIL and 3.549 (95% CI 1.089–11.566) in DBIL, respectively. In men, there was no association between the bilirubin tertiles and the risk of END (shown in Table 4).

**Table 4:** Adjusted odds ratio for END according to serum bilirubin levels.

Subgroup		END outcome *n* (%)	Unadjusted OR (95% CI)	*p-*value	Adjusted OR (95% CI)	*p-*value
**Women**						
TBIL	T1	12 (31.6)	1.0(reference)		1.0(reference)	
	T2	18 (48.6)	2.053 (0.802–5.254)	0.134	2.637 (0.861–8.081)	0.090
	T3	21 (67.7)	4.550 (1.645–12.584)	0.004^*^	5.240 (1.496–18.347)	0.010^*^
	p for trend		2.130 (1.282–3.539)	0.004^*^	2.303 (1.232–4.308)	0.009^*^
DBIL	T1	11 (30.6)	1.0(reference)		1.0(reference)	
	T2	20 (57.1)	3.030 (1.143–8.036)	0.026^*^	3.890 (1.243–12.169)	0.020^*^
	T3	20 (57.1)	3.030 (1.143–8.036)	0.026^*^	3.549 (1.089–11.566)	0.036^*^
	p for trend		1.730 (1.067–2.805)	0.026^*^	1.897 (1.055–3.414)	0.033^*^
IDBL	T1	12 (33.3)	1.0(reference)		1.0(reference)	
	T2	18 (51.4)	2.118 (0.812–5.525)	0.125	2.896 (0.933–8.987)	0.066
	T3	21 (60.0)	3.000 (1.139–7.900)	0.026^*^	3.006 (0.905–9.986)	0.072
	p for trend		1.730 (1.067–2.805)	0.026^*^	1.723 (0.947–3.134)	0.075
**Men**						
TBIL	T1	45 (56.3)	1.0(reference)		1.0(reference)	
	T2	56 (63.6)	1.361 (0.733–2.529)	0.329	1.643 (0.844–3.200)	0.144
	T3	41 (58.6)	1.100 (0.575–2.104)	0.774	1.106 (0.545–2.244)	0.780
	p for trend		1.056 (0.762–1.465)	0.743	1.069 (0.748–1.528)	0.713
DBIL	T1	48 (59.3)	1.0(reference)		1.0(reference)	
	T2	45 (57.7)	0.938 (0.499–1.762)	0.841	0.944 (0.473–1.885)	0.871
	T3	49 (62.0)	1.123 (0.595–2.118)	0.720	1.051 (0.526–2.099)	0.888
	p for trend		1.059 (0.772–1.452)	0.724	1.026 (0.726–1.449)	0.885
IDBL	T1	47 (58.0)	1.0(reference)		1.0(reference)	
	T2	48 (61.5)	1.157 (0.614–2.183)	0.652	1.304 (0.663–2.563)	0.441
	T3	47 (59.5)	1.062 (0.566–1.994)	0.850	1.094 (0.552–2.168)	0.797
	p for trend		1.031 (0.752–1.415)	0.848	1.050 (0.745–1.481)	0.781

95% CI, 95% confidence interval. Adjusted for age, NIHSS on admission, hypertension, diabetes, hyperglycemia, AF, smoking, TOAST types, UA, HDL, LDL. ^*^p < 0.05.

Multivariate logistic regression showed that DBIL level was an independent predictor of END in women (OR 1.717, 95% CI 1.106–2.666) after adjusting for variables with *p* < 0.1 on univariate analysis and TOAST types (shown in Table 5). In men, bilirubin levels were not associated with the risk of END (shown in Supplementary Table S1).

**Table 5:** Logistic regression of clinical factors affecting the occurrence of END in women.

Variables in women	Univariate		Multivariate	
	OR (95% CI)^a^	*p-*value	OR (95% CI)	*p-*value
Age	1.000 (0.970–1.030)	0.985		
Hypertension	1.110 (0.457–2.694)	0.818		
Systolic BP	1.010 (0.992–1.028)	0.295		
Diastolic BP	0.999 (0.962–1.038)	0.971		
Diabetes	1.820 (0.837–3.957)	0.131		
Hyperlipidemia	2.002 (0.923–4.344)	0.079	2.194 (0.930–5.176)	0.073
Coronary heart disease	0.556 (0.173–1.785)	0.324		
Smoking history	1.656 (0.265–10.338)	0.589		
Drinking history	2.204 (0.194–25.071)	0.524		
Stroke or TIA	0.715 (0.292–1.749)	0.462		
Atrial fibrillation	0.118 (0.014–0.976)	0.047^*^	0.095 (0.010–0.915)	0.042^*^
NIHSS score on admission	1.055 (0.948–1.175)	0.329		
TOAST type		0.806		
Large-artery atherosclerosis	Reference			
Cardioembolism	0.500 (0.041–6.082)	0.587		
Small-artery occlusion	1.000 (0.424–2.359)	1.000		
Undetermined cause	0.571 (0.139–2.342)	0.437		
TC	1.179 (0.834–1.666)	0.351		
HDL	1.520 (0.377–6.128)	0.556		
LDL	1.280 (0.817–2.005)	0.282		
Lp(a)	0.999 (0.997–1.001)	0.263		
TG	1.130 (0.676–1.888)	0.642		
HbA1c	1.050 (0.861–1.280)	0.631		
hs-CRP	1.000 (0.949–1.053)	0.994		
D-dimer	1.191 (0.754–1.880)	0.454		
UA	0.996 (0.991–1.000)	0.067	0.995 (0.990–1.000)	0.050
TBIL	1.069 (0.991–1.153)	0.083		
DBIL	1.519 (1.020–2.263)	0.040^*^	1.717 (1.106–2.666)	0.016^*^
IBIL	1.074 (0.983–1.173)	0.113		

95% CI, 95% confidence interval; BP, blood pressure; TIA, transient ischemic attack; TC, total cholesterol; TG, total triglycerides; HDL, high-density lipoprotein; LDL, low-density lipoprotein; Lp(a), lipoprotein a; HbA1c, glycosylated hemoglobin; hs-CRP, high sensitivity C-active protein; UA, uric acid; TBIL, total bilirubin; DBIL, direct bilirubin; IBIL, indirect bilirubin. ^*^p < 0.05.

In order to further evaluate the incremental predictive value of different bilirubin subtypes for END in women, we added TBIL, DBIL and IBIL to model 1 (conventional model) and model 2 (from logistic regression results) respectively and tested the effects of different models. Based on AIC, C-statistic, IDI and NRI, we found that DBIL was superior to TBIL and IBIL in prediction of END, with lower AIC and higher IDI (Shown in Table 6).

**Table 6:** Incremental predictive value of different bilirubin subtypes for END in women.

Model	AIC	*p-*value	C-statistic	*p-*value	IDI (relative IDI)	*p-*value	NRI	*p-*value
Model 1	157.3987	Ref.	0.7127 (0.6137–0.8116)	Ref.	Ref.	Ref.	Ref.	Ref.
Model 1 +TBIL	158.9821	0.5187	0.7023 (0.6026–0.8020)	0.3759	0.0027 (0.0569)	0.6287	0.0271	0.8892
Model 1 +DBIL	154.6813	0.0299^*^	0.7251 (0.6290–0.8213)	0.7220	0.0410 (0.3203)	0.0442^*^	0.2531	0.1929
Model 1 +IBIL	159.0595	0.5603	0.7130 (0.6144–0.9117)	0.9741	0.0033 (0.0293)	0.5242	0.3373	0.0828
Model 2	143.1433	Ref.	0.6553 (0.5502–0.7603)	Ref.	Ref.	Ref.	Ref.	Ref.
Model 2 +TBIL	144.8260	0.5732	0.6563 (0.5516–0.7611)	0.9275	0.0029 (0.0711)	0.5785	0.0663	0.7330
Model 2 +DBIL	139.3811	0.0164^*^	0.7020 (0.6020–0.8020)	0.2326	0.0468 (0.4762)	0.0296^*^	0.3287	0.0909
Model 2 +IBIL	144.7274	0.5190	0.6734 (0.5697–0.7771)	0.1821	0.0045 (0.0808)	0.4476	0.1497	0.4412

AIC, Akaike’s information criterion; IDI, integrative discrimination improvement; NRI, net reclassification improvement; Ref., reference. Model 1: conventional model: age, NIHSS on admission, hypertension, diabetes, hyperglycemia, AF, smoking, TOAST types, UA, HDL, LDL. Model 2: hyperglycemia, AF, UA. ^*^p < 0.05.

## 4 Discussion

Our study demonstrated that there were gender differences in the relationship between bilirubin and END. DBIL level was independently associated with END occurrence in women, not in men. In addition, DBIL had greater incremental predictive value for END than TBIL and IBIL. To the best of our knowledge, this is the first study to investigate the relationship of different subtypes of bilirubin with END in different genders.

So far the studies of bilirubin in stroke still yield conflicting results. There were some studies showing insignificant relationship between bilirubin and the risk of stroke. Jorgensen et al. showed that bilirubin was not an independent risk factor after adjusting for other traditional risk factors (11). However, a large number of studies supported a protective role of bilirubin in stroke occurrence. A study of 13,214 individuals reported that a 1.71 μmol/L increment in bilirubin level was associated with a 9% reduced odds of stroke (12). The meta-analysis by Kunutsor et al. reported that the pooled relative risk for stroke was 0.93 (95% CI 0.88–0.98) per 1-SD increase in total bilirubin levels (13). Another meta-analysis also demonstrated that a 15 μmol/L increment of bilirubin level was associated with an 18% lower risk of stroke (RR 0.82, 95% CI 0.58–0.99) (14). Using Mendelian randomization analysis Choi et al. found an inverse causal association between serum bilirubin levels and total stroke risk (OR 0.481, 95% CI 0.234–0.988) (15). In a large cohort of 19,906 Chinese hypertensive patients, an inverse association was also found between TBIL, DBIL and the risk of first ischemic stroke (16).

In addition to stroke occurrence, bilirubin could also be associated with functional outcome in stroke, whereas the results were also inconsistent. Some reported a positive correlation of bilirubin with good prognosis. Perlstein et al. (12) found an association of higher serum total bilirubin with improved stroke outcomes and Sheng et al. (17) showed that decreased TBIL predicted END in AIS patients. Duan et al. (18) found that in patients with mild stroke (NIHSS ≤5), elevated bilirubin after AIS suggested a good prognosis (3-month mRS ≤ 2). Meanwhile, some researchers reported a null relationship of bilirubin with stroke prognosis. For example, Pineda et al. (19) found no independent relationship between TBIL and functional outcome at discharge after adjusting for confounders. Xu et al. (20) showed that serum bilirubin was not significantly associated with short-term clinical outcomes (NIHSS ≥ 10 at discharge) or in-hospital death. However, there were also studies supporting the association of high bilirubin with poor prognosis. Peng et al. (21) showed that increased DBIL before thrombolysis was associated with poor functional outcome (3-month death and major disability; OR 3.228, 95% CI 1.595–6.535). Kurzepa et al. (22) observed a negative correlation between TBIL level and 3-month Barthel index (r = −0.5, *p* < 0.01). Ouyang et al. (23) reported that elevated bilirubin was associated with poor functional outcome at 3 months and 1 year in patient with AIS or TIA. Sagheb Asl et al. (24) showed that TBIL, DBIL and IBIL levels were significantly associated with mortality in the acute phase of ischemic stroke.

Our results supported that higher DBIL was an independent predictor of END in AIS. There are two possible explanations. The first explanation is that although bilirubin is an endogenous antioxidant, it could also be a marker of oxidative stress and higher levels of bilirubin might reflect stronger oxidative reaction in the ischemic state. A study by Cui et al. (25) showed that depletion of glutathione (GSH) could induce heme oxygenase (HO) −1 gene expression, which was the key enzyme of bilirubin production. Another possible explanation is the double-edged role of bilirubin. Bilirubin could be protective and beneficial at a low concentration, but cytotoxic at a pathological concentration. A meta-analysis proposed that 10 μmol/L could be a cut-point of TBIL for discrimination of cardiovascular risk (26). Creeden et al. (27) proposed a hypothetical curve of bilirubin in the general population based on the reported studies, in that bilirubin < 10 μmol/L represents hypobilirubinemic states and bilirubin of 25–50 μmol/L represents mild hyperbilirubinemic states. However, this classification did not take the gender difference and pathological states into account. A recent study suggests that ischemic insults triggers the release of endogenous bilirubin from injured cells, which activates the transient receptor potential melastatin 2 (TRPM2) channels and aggravates Ca^2+^-dependent brain injury (28). This vicious cycle of ischemic injury might partly contribute to the transformation of bilirubin from benefit to toxicity in ischemic stroke.

Our results also showed gender differences in the association of bilirubin with END occurrence. Previous studies have reported the difference in the levels of bilirubin between men and women (29), which was also observed in our study. The reason why the bilirubin is higher in men remains indecisive, possibly due to the serum estrogen, iron storage, life style or other undefined reasons (30). There are some studies showing the gender difference of the association of bilirubin with stroke or other diseases. The meta-analysis of Zhao et al. (31) showed that TBIL level in males correlated with stroke risk, but not in females. Kim et al. (32) showed that bilirubin was protective against stroke only in men, not in women. In other diseases, Han et al. (33) indicated that bilirubin had protective function against diabetes mellitus (DM) and chronic kidney disease originated from DM only in women. Park et al. (29) reported that TBIL was inversely associated with leukoaraiosis in Korean women, not in men. However, the study of Endler et al. (34) indicated an association between bilirubin and coronary disease only in males. A recent study in Chinese population demonstrated that the incidence of fundus arteriosclerosis was positively correlated with serum TBIL level in males, not in females (35). The discrepancy and underlying mechanisms need to be further investigated. The difference of disease and race might contribute to the discrepancy.

Our results further compared the predictive value of different bilirubin subtypes and found that DBIL had greater incremental predictive value for END occurrence than TBIL and IBIL. There were some studies investigating the effects of TBIL, DBIL (6, 16, 31, 36) and IBIL (23, 24) in different diseases, but very few studies compared the predictive value of bilirubin subtypes. Our study is consistent with the study of Peng et al., in which DBIL was found to have greater predictive value of functional outcome after thrombolysis (21). However, Peng’s study focused on patients after thrombolysis and did not take the gender into account. It is speculated that DBIL is weakly bound to albumin and might be more active to act on the target organs than IBIL, which makes DBIL more related to pathological states. More evidence is needed to validate this speculation.

In addition to bilirubin, our results also found other independent predictors of END. In women, after multi-variate adjustment by the backward likelihood regression, four factors were left in the model, including hyperlipidemia history, AF history, UA and DBIL. Interestingly, UA also has both the antioxidant and toxic properties, similar to bilirubin. The role of UA in stroke also remains controversial. Some studies suggested that elevated uric acid could confer protection against neurologic deficit in stroke, while others held a point that elevated UA was injurious rather than neuroprotective (37). In our study, UA level tended to be inversely related with END occurrence, suggesting a protective role. The role of UA in stroke remains to be further validated.

Our study has the following implications. First, we demonstrated that the serum bilirubin level was positively associated with END occurrence and could be a predictive biomarker of END. Our study provides more insight into the risk factors of END and the complex role of bilirubin in stroke. Second, we found a gender difference in the effect of elevated bilirubin on END, although the underlying mechanisms remain unclear. Multiple factors might possibly contribute to the difference between men and women, such as serum estrogen, iron storage, heme oxygenase and life style (drinking, smoking and diet, etc.). Regardless, lower baseline bilirubin levels in women may indicate lower oxidative stress or a stronger antioxidant defense system. In other words, the antioxidant system in women tends to be more stable in normal conditions. Our study implied that the abnormally elevated bilirubin in the state of diseases should be taken more seriously in women. Third, we reported that among the different subtypes of bilirubin, elevated DBIL may be more predictive of END. Instead of analyzing all bilirubin subtypes, it could reduce the costs to analyze DBIL, especially when repeated analysis was needed over the course of disease.

There are some limitations in our study. First, our study is a retrospective study, which was not the best way to investigate the causal relationships. However, in our study, the cohort was recruited from prospectively maintained registries, which makes the selection bias less likely to affect our major findings. Second, the study is a single-center study with small sample size. Prospective studies with larger sample size are needed in the future. Third, our study is a cross-sectional study which only analyzed the association of serum bilirubin level on admission with neurological deterioration. Since there are individual differences in the baseline serum bilirubin, the changing trends of serum bilirubin levels might be more reflective of the intensity of oxidative stress and disease development. Future researches could further investigate the association of stroke prognosis with serum bilirubin levels at different time points to find out whether the change of bilirubin is a better indicator of stroke prognosis. Last, the mechanisms underlying gender differences of bilirubin remain to be elucidated. In our study, information concerning menopausal status, oral contraception and diet was not collected. Future research is warranted to further clarify the gender difference in the effects of bilirubin.

## 5 Conclusion

There are gender differences in the relationship between bilirubin and END, and DBIL level was positively associated with END occurrence in women, not in men. DBIL had greater incremental predictive value for END than TBIL and IBIL.

## Data availability statement

The raw data supporting the conclusions of this article will be made available by the authors, without undue reservation.

## Ethics statement

The studies involving humans were approved by the Ethics Committee of Putuo Hospital, Shanghai University of Traditional medicine. The studies were conducted in accordance with the local legislation and institutional requirements. The participants provided their written informed consent to participate in this study.

## Author contributions

MS: Data curation, Formal analysis, Writing – review & editing. YW: Data curation, Formal analysis, Writing – review & editing. JC: Data curation, Writing – review & editing. GL: Conceptualization, Writing – review & editing. QX: Conceptualization, Formal Analysis, Writing – original draft, Writing – review & editing.
