# Supplementary material for: Higher baseline serum bilirubin levels are associated with increased risk of early neurological deterioration in women with acute ischemic stroke
Source: Front Neurol. 2024 Apr 8;15:1381055. doi: 10.3389/fneur.2024.1381055 (PMC11033363; doi:10.3389/fneur.2024.1381055)
Supplement: Supplementary file 1 [file Table_1.DOCX]

***Supplementary Materials***

**1. Supplementary Tables**

**Table S1. Logistic regression of clinical factors affecting the occurrence of END in men.**

| **Variables**  **in men** | **Univariate** |  | **Multivariate** |  |
| --- | --- | --- | --- | --- |
|  | **OR (95% CI)^a^** | ***p* value** | **OR (95% CI)** | ***p* value** |
| Age | 1.005(0.980-1.031) | 0.681 |  |  |
| Hypertension | 0.612(0.312-1.201) | 0.153 |  |  |
| Systolic BP | 1.003(0.990-1.016) | 0.649 |  |  |
| Diastolic BP | 1.018(0.995-1.042) | 0.127 |  |  |
| Diabetes | 0.998(0.593-1.678) | 0.993 |  |  |
| Hyperlipidemia | 1.166(0.690-1.970) | 0.567 |  |  |
| Coronary heart disease | 1.699(0.712-4.057) | 0.232 |  |  |
| Smoking history | 0.894(0.522-1.531) | 0.682 |  |  |
| Drinking history | 0.890(0.518-1.530) | 0.673 |  |  |
| Stroke or TIA | 1.425(0.791-2.566) | 0.238 |  |  |
| Atrial fibrillation | 0.280(0.084-0.938) | 0.039^*^ | 0.277(0.083-0.926) | 0.037^*^ |
| NIHSS score on admission | 1.069(0.972-1.175) | 0.169 |  |  |
| TOAST type |  | 0.143 |  |  |
| Large-artery atherosclerosis | Reference |  |  |  |
| Cardioembolism | 0.157(0.031-0.812) | 0.027^*^ |  |  |
| Small-artery occlusion | 0.855(0.473-1.544) | 0.603 |  |  |
| Undetermined cause | 0.636(0.264-1.531) | 0.312 |  |  |
| TC | 1.220(0.946-1.573) | 0.126 |  |  |
| HDL | 2.375(0.689-8.189) | 0.171 |  |  |
| LDL | 1.230(0.890-1.698) | 0.210 |  |  |
| Lp(a) | 1.000(0.999-1.001) | 0.999 |  |  |
| TG | 0.942(0.730-1.215) | 0.644 |  |  |
| HbA1c | 0.974(0.839-1.132) | 0.735 |  |  |
| hs-CRP | 1.016(0.981-1.051) | 0.374 |  |  |
| D-dimer | 0.819(0.552-1.217) | 0.324 |  |  |
| UA | 0.997(0.995-1.000) | 0.092 |  |  |
| TBIL | 1.007(0.971-1.045) | 0.693 |  |  |
| DBIL | 1.081(0.896-1.303) | 0.417 |  |  |
| IBIL | 1.006(0.964-1.050) | 0.784 |  |  |

Abbreviation: END: early neurological deterioration; OR: odds ratio; 95% CI: 95% confidence interval; BP: blood pressure; TIA: transient ischemic attack; TC: total cholesterol; TG: total triglycerides; HDL: high-density lipoprotein; LDL: low-density lipoprotein; Lp(a):lipoprotein a; HbA1c: glycosylated hemoglobin; hs-CRP: high sensitivity C-active protein; UA: uric acid; TBIL: total bilirubin; DBIL: direct bilirubin; IBIL: indirect bilirubin. ^*^ *p* < 0.05.
